# Prospective memory, retrospective memory, and individual differences in cognitive abilities, personality, and psychopathology

**DOI:** 10.1371/journal.pone.0193806

**Published:** 2018-03-27

**Authors:** Bob Uttl, Carmela A. White, Kelsey Cnudde, Laura M. Grant

**Affiliations:** Psychology Department, Mount Royal University, Calgary, Alberta, Canada; University of Akron, UNITED STATES

## Abstract

Although individual differences in processing speed, working memory, intelligence, and other cognitive functions were found to explain individual differences in retrospective memory (RetM), much less is known about their relationship with prospective memory (ProM). Moreover, the studies that investigated the relationship between ProM and cognitive functions arrived to contradictory conclusions. The relationship between ProM, personality, and psychopathology is similarly unsettled. Meta-analytic reviews of the relationships of ProM with aging and personality suggest that the contradictory findings may be due to widespread methodological problems plaguing ProM research including the prevalent use of inefficient, unreliable binary measures; widespread ceiling effects; failure to distinguish between various ProM subdomains (e.g., episodic ProM versus vigilance/monitoring); various confounds; and, importantly, small sample sizes, resulting in insufficient statistical power. Accordingly, in a large scale study with nearly 1,200 participants, we investigated the relationship between episodic event-cued ProM, episodic RetM, and fundamental cognitive functions including intelligence, personality, and psychopathology, using reliable continuous measures of episodic event-cued ProM. Our findings show that (a) continuous measures of episodic event-cued ProM were much more reliable than binary measures, (b) episodic event-cued ProM was associated with measures of processing speed, working memory, crystallized and fluid intelligence, as well as RetM, and that such associations were similar for ProM and RetM, (c) personality factors did not improve prediction of neither ProM nor RetM beyond the variance predicted by cognitive ability, (d) symptoms of psychopathology did not improve the prediction of ProM although they slightly improved the prediction of RetM, and (e) participants' sex was not associated with ProM but showed small correlations with RetM. In addition to advancing our theoretical understanding of ProM, our findings highlight the need to avoid common pitfalls plaguing ProM research.

## Introduction

Prospective memory (ProM) allows us to make plans, retain them, and bring them back to one's consciousness at the right time and place [1–3]. ProM is used for a variety of everyday tasks including remembering to buy groceries en route home, picking up a child from daycare, keeping appointments, watching a bathtub so it does not overflow, taking medication at bedtime, and paying bills by the deadline. ProM is divided into several subdomains [1,2]: episodic ProM or ProM proper, vigilance/monitoring, and habitual ProM. Episodic ProM brings back to consciousness a previously formed plan at the right time or place, in response to appropriate cues (e.g., remembering to buy groceries en route home when seeing the grocery store sign). In contrast, vigilance/monitoring maintains the plan in consciousness from the formation of the plan until the right time or place for its performance (e.g., watching a bathtub so that it does not overflow). Habitual ProM is similar to episodic ProM, but the time and place for performing the plan repeats regularly with enough time between repetitions for the plan to leave consciousness (e.g., taking medication everyday at bedtime). Moreover, ProM may be cued by events (event-cued or event-based) or by time (time-cued or time-based). In this study, we focus on episodic event-cued ProM.

Although individual differences in processing speed, working memory, intelligence, and other cognitive functions were found to explain individual differences in retrospective memory (RetM), much less is known about their relationship with ProM. Moreover, the studies that have investigated the relationship between ProM, intelligence, and other cognitive functions have arrived to opposite conclusions. For example, Maylor [4–6] reported no significant correlations between ProM measures and verbal intelligence tests including the Mill Hill Vocabulary Test [7] and the vocabulary subtest of the Wechsler Adult Intelligence Scale–Revised [8]. Others have similarly found no relationships between ProM measures and verbal intelligence measures [9–13]. In contrast, other studies found small to moderate correlations between ProM and verbal intelligence [14–18]. Similarly, inconsistent findings have been reported for fluid intelligence, working memory, processing speed, and RetM measures, even within these same studies.

Conflicting findings have also been reported for the relationships between ProM measures and measures of personality. Although Searleman noted that “people vary tremendously in their ability to successfully carry out many types of prospective memory tasks–some are astonishingly proficient at such tasks, whereas others are absolutely terrible at them,” [19] his and subsequent research found only weak and inconsistent relationships between ProM and personality factors [17,19]. Our meta-analysis of the relationship between personality and ProM found only weak correlations (.09 to .10) between ProM and three personality factors: openness, conscientiousness, and agreeableness [17]. However, the meta-analysis was based on a mix of episodic ProM and vigilance/monitoring studies, and because of the insufficient number of studies, we could not determine if the relationship between ProM and personality varied by ProM subdomain. Our subsequent large scale experimental study showed that episodic ProM was not related to any of the Big Five personality factors, whereas vigilance/monitoring was correlated with extroversion and openness [17]. Nevertheless, even our relatively large sample study (n = 283 for episodic ProM) did not have sufficient statistical power to detect correlations of .10 as suggested by the meta-analysis.

What may be the reasons for the inconsistent findings among the relationships between ProM and other cognitive functions and personality? We have previously examined similarly contradictory findings about age-related differences in ProM [2,17,20]. While some researchers concluded that there were no age related declines in ProM others found large age declines. In our reviews, we found a number of reasons for these inconsistent findings. First, ProM was typically assessed using binary success/failure measures. One natural consequence of such measurement is that ProM measures are imprecise, inefficient, and not reliable. In turn, binary measures substantially underestimate the true population correlation between ProM and aging. Further, such underestimation was more severe as the proportion of success to failures became more extreme. Second, a large proportion of ProM studies suffered from severe ceiling effects, with a large proportion of participants in such studies obtaining perfect or nearly perfect scores. In turn, the ceiling effects artificially reduced the observed correlations between ProM and aging. In fact, the strongest reported and independently replicated relationship involving ProM to date is *r* = .67 between the magnitude of age related decline in ProM and the degree to which the ProM performance was limited by ceiling effects [2,16,21,22]. Third, although many studies acknowledge the distinction between episodic ProM and vigilance/monitoring, it is rarely made explicit and readers must carefully read through method sections of various studies to determine whether a study examined episodic ProM or vigilance/monitoring. Importantly, we previously found that both age declines and personality correlations vary depending on ProM subdomain. Fourth, many previous studies confounded a variable of interest–age–with other variables, for example, intelligence. Finally, and equally important, most of the studies on ProM are based on small samples and do not have sufficient statistical power to detect even moderate correlations. We suspect the same methodological issues may be responsible for inconsistent findings among correlations between event-cued ProM, cognitive abilities, and personality.

Recently, there has also been an interest in the relationship between ProM, depression [23], anxiety [23], and other symptoms of psychopathology such as obsessive compulsive disorder (OCD) [24–26]. Depression as well as anxiety are presumed to limit the amount of processing resources, and thus, reduce ProM performance. However, research findings are conflicting, with some studies finding negative correlations between ProM and depression and anxiety [9,23,27] and other studies finding no such relationships [23,28]. In contrast, theories about the relationship between ProM and OCD suggest that symptoms of OCD may cause ProM failures but also be caused by ProM failures. For example, symptoms of OCD combined with the awareness of past ProM failures may trigger increases in OCD symptoms such as compulsive checking [29]. But OCD associated with deficits in working memory and executive functions, may also cause ProM failures. Not surprisingly, some studies found a relationship between ProM and OCD, whereas other studies found no such relationships [29]. When reviewing the literature on ProM and compulsive checking, Cuttler and Graf pointed to several possible explanations including small sample sizes, failures to distinguish between episodic ProM and vigilance/monitoring, and a number of confounds when OCD groups were compared to healthy controls [29].

Finally, it has been suggested that non-cognitive factors (i.e., personality, lifestyle, etc.) are more influential than cognitive factors (i.e., processing speed, intelligence) in determining prospective memory performance [30]. In contrast, cognitive factors were found to be far more influential than personality factors in determining retrospective memory performance. However, to our knowledge, only one previous study examined relative importance of personality and lifestyle variables vs. cognitive variables on prospective memory performance but its results were different for different episodic prospective memory tasks and ultimately inconclusive [30]. Possible reasons for inconsistent findings were use of unreliable binary measures of prospective memory and prevalent ceiling effects on some but not other prospective memory tasks. Moreover, the authors noted that their study design did not allow any direct comparison of predictive power of personality and lifestyle vs. cognitive factors on episodic prospective vs. episodic retrospective memory [30].

Accordingly, the main objective of our study was to investigate the relationship between episodic event-cued ProM versus episodic RetM and fundamental cognitive functions, personality, and symptoms of psychopathology, using reliable continuous measures of ProM and a large sample of participants to ensure sufficient statistical power to detect even small (*r* = .10) relationships suggested by our meta-analysis of the relationships between ProM and personality [17]. The power analyses indicated that to detect *r* = .10, with power of .80 and two-tailed alpha of .05, the required sample size is *N* = 783 participants; to detect the same effect with power of .90 the required sample size is *N* = 1,047 participants; and to detect the same effect with power of .95, the required sample size is *N* = 1,294 participants. We aimed for statistical power of at least .90 to detect *r* = .10

There were five specific aims of the current study. The first aim was to examine the distribution, intercorrelations, and the composite reliability of two continuous measures of event-cued ProM. The second aim was to examine the relationships between event-cued ProM versus RetM and a variety of cognitive abilities including crystallized intelligence, fluid intelligence, processing speed, and working memory. The third aim was to examine the relationship between event-cued ProM versus RetM and personality. The fourth aim was to examine the relationship between even cued ProM versus RetM and depression, anxiety, and other symptoms of psychopathology. The fifth aim was to examine whether these relationships differ depending on participants' sex. And the final, sixth aim, was to examine relative contribution of personality vs. cognitive factors to performance on episodic prospective vs. episodic retrospective memory measures.

## Method

### Participants

Participants were 1170 undergraduate student volunteers, 79.9% women and 20.1% men, (age *M* = 21.43 years, *SD* = 5.24 years). The majority of participants spoke English as their first language (80.0%). The study was approved by Mount Royal University Human Research Ethics Board and all participants gave written consent to participate in the study. The study took approximately 2 hours and each participant received two course participation credits.

### Measurement instruments

As part of a larger study, participants were administered several objective tests of ProM and RetM, measures of crystallized and fluid intelligence, working memory, processing speed, and personality. Table 1 shows the sequence of all questionnaires and tasks completed by the participants. Other questionnaires including the inner speech questionnaires and a course interest survey were included to insert a delay between ProM instructions and the start of the ongoing tasks as well as for other unrelated reasons that are not analyzed in this paper.

**Table 1:** Sequence of all instruments and tasks completed by participants, with the approximate time required to complete each task.

Task	Approximate time required (min)
Demographics	3
Inner Speech Report	10
Digit Span Backwards	5
Digit Symbol Coding	3
VLT/C20	10
SILS/R	10
Self-Talk Scale	5
ProM Instructions 1	2
Varieties of Inner Speech	5
SCL-90R	15
Words/A40 w/embedded ProM cues	15
ProM Instructions 2	2
Self-Regulation Questionnaire	5
Course Interest Survey	10
IPIP100 w/embedded ProM cues	15
SCS	5

*Note*. VLT/C20 = Verbal Learning Test Categorized 20, SILS/R = Shippley's Institute for Living Scale Reasoning Test, SCL-90R = Symptoms Checklist 90 Revised, IPIP100 = International Personality Item Pool NEO, SCS = Self Control Scale.

#### Continuous measures of event-cued ProM (ProM/C)

The continuous measures of event-cued ProM developed for this study were patterned after continuous measures previously used by Uttl and his colleagues [15,16,31]. Participants' ProM was assessed on two occasions within the session to allow assessment of test-retest reliability. For each assessment, participants were instructed to circle any and all occurrences of the ProM cue–the word “content” (first assessment) and the word “close” (second assessment)–as they worked through the session. The specific instructions for the first assessment were:

We want to examine your ability to do something in the future. Thus, if you encounter the word *content* at any point during this experiment, please circle it. You will not be reminded again but it is important that you circle any and all occurrences of the word *content*. Please copy the following sentence below in your hand writing so that we are sure you did not miss these instructions: “I am to circle all occurrences of the word *content*.”

The instructions for the second assessment were identical except that the word *content* was replaced with the word *close*. Following these instructions, participants worked through the tasks.

For the first assessment, eight ProM cues were embedded within a word knowledge multiple choice test–Words/A40 (see below)–extended with 18 fillers and eight items that included ProM cues. Each page included six multiple choice items and one ProM cue appeared on each page starting with page four until the last page, page 11. Critically, the ProM cue became progressively larger and more intrusive on each successive page. The smallest cue was 12 points (identical to the surrounding text font size) and the largest cue was 28 points. If an examinee detected the very first cue, he or she obtained a score of eight. If the examinee detected the last cue, he or she obtained score of 1, and if the examinee did not detect any cues, he or she obtained score of 0. Thus, each participant's test score was determined solely by the first cue to which the participant responded.

For the second assessment, eight ProM cues were embedded within a personality inventory designed to measure the Big Five–IPIP 100 (see below)–extended with 33 fillers and eight items that included ProM cues. Both fillers and items containing ProM cues were drawn from the IPIP database. Thus, on each assessment, participants encountered the cues within an 11 page personality inventory. The first page contained the standard instructions for the personality inventory and each of the subsequent 10 pages had 15 personality items on it except the last page that had only six items. One ProM cue appeared on each page starting with page five. The ProM cues increased in size and the test was scored the same way as on the first assessment.

#### Binary measure of event-cued ProM (ProM/B)

To compare performance on binary vs. continuous measures, for each ProM assessment above, we also calculated a binary success/failure ProM measure based solely on participants' performance on the first shown ProM cue. If a participant responded to the very first cue, he or she obtained a score of 1. If the participant did not respond to the first cue, he or she received a score of 0.

#### Words/A40

The Words/A40 is a 40-item multiple choice test designed to assess examinees' verbal knowledge [15]. Each item consists of a target word and four other words out of which one word is similar in meaning. Each item is scored as correct (1 point), incorrect (0 points), or not answered (0.25 points to correct for a failure to guess). The test score is the average across all items or the proportion of items correctly answered corrected for failure to guess.

#### International personality Item Pool NEO (IPIP 100)

The IPIP 100 [32] is a 100-item self-report personality inventory designed to measure the Big Five using items from the International Personality Item Pool (IPIP). Examinees rate how accurately each statement describes them using the following response scale: 1 = *Very inaccurate*, 2 = *Moderately inaccurate*, 3 = *Neither inaccurate nor accurate*, 4 = *Moderately accurate*, and 5 = *Very accurate*. Similarly to NEO PI-R, we calculated each participant's personality dimension scores as an average across the relevant items.

#### Shippley's Institute for Living Scale Reasoning test (SILS/R)

The SILS/R [33] is a 20-item test designed to assess examinees' reasoning skills. Examinees are asked to complete various sequences with the next item in the sequence, for example, if they are given the sequence of 2 4 16 __ they are expected to fill in 144 (2x2 = 4, 4x4 = 16, 16x16 = 144). The score is the proportion of the number of sequences correctly completed out of 20.

#### Verbal Learning Test Categorized 20 (VLT/C20)

The VLT/C20 [15] is a measure of explicit episodic RetM patterned after the Rey Auditory Verbal Learning Test [34] but five items are selected from each of the four categories: animals, clothing, vegetables, and kitchen items. The test consists of a series of three study-test trials on one set of 20 words selected from the four categories. On each trial, an examinee listened to a list of 20 words read by an experimenter and were required to write down as many words as they could remember, in any order, after the experimenter had completed reading the list. For each trial, participants were given 90 seconds for recall. The scores on each trial as well as the average of the three trials could range from 0 to 20.

#### Wechsler IV Digit Symbol Coding (DS Coding)

The DS Coding [35] is primarily a measure of processing and motor speed. Examinees are presented with a two row digit-symbol translation table with numbers from one to nine in the top row and the symbols corresponding to each number in the bottom row. Below the table, examinees are presented with rows of numbers and their task is to draw the corresponding symbols below each of the numbers, working as fast as they can, for 120 seconds. The DS Coding score is the number of correctly placed symbols with a maximum score of 135.

#### Wechsler IV Digit Span Backward (DS Backward)

The DS Backward [35] measures working memory. Examinees are read sequences of numbers and their task is to repeat back each sequence backwards. For example, if an examinee hears 3–5–4, he or she needs to repeat back 4–5–3. The DS Backward score is the length of the largest sequence that the examinee repeats back successfully.

#### Symptoms Checklist 90 Revised (SCL90R)

The SCL90R [36] is a 90-item questionnaire assessing psychological problems and symptoms of psychopathy. It includes nine primary dimensions: Somatization (SOMA), Obsessive-Compulsive (O-C), Interpersonal Sensitivity (I-S), Depression (DEP), Anxiety (ANX), Hostility (HOS), Phobic Anxiety (PHOB), Paranoid Ideation (PAR), Psychoticism (PSY). It also provides three global indexes: Global Severity Index (GSI) measures overall psychological distress; Positive Symptom Distress Index (PSDI) measures intensity of symptoms; and Positive Symptom Total (PST) is the total number of self-reported symptoms.

#### Course interest survey

The Course Interest Survey [37] consisted of 44 titles and descriptions of all psychology courses offered in the 2012–2013 Mount Royal University calendar except the two introductory psychology first year courses that participants were registered in. The participants rated their interest in taking each course using a 5-point scale ranging from 1 = *Not at all interested* to 5 = *Very interested*.

#### Self Talk Scale (STS)

The STS [38] is a 22-item questionnaire measuring the frequency of both inner and private speech. Examinees indicate the frequency with which they engage in various self-talk using the 5-point scale: 1 = N*ever*, 2 = *Seldom*, 3 = *Sometimes*, 4 = *Often*, and 5 = *Very often*.

#### Inner speech report

The Inner Speech Report required participants to think about what they talk to themselves about. They were asked to write down as many things as they talk to themselves about as they could recall. The responses were coded for the number of inner speech instances produced and also classified into specific categories by content and function.

#### Varieties of Inner Speech Questionnaire (VISQ)

The VISQ [39] is an 18-item questionnaire designed to measure the phenomenological properties of inner speech including dialogic inner speech, condensed inner speech, other people in the inner speech, and evaluative/motivational inner speech. Examinees responded to each statement using a 6-point scale: 6 = *Certainly applies to me*, 5 = *Possibly applies to me*, 4 = *If anything*, *applies to me slightly*, 3 = *If anything*, *slightly does not apply to me*, 2 = *Possibly does not apply to me*, and 1 = *Certainly does not apply to me*.

#### Self Regulation Questionnaire (SRQ)

The SRQ [40] is a 63-item questionnaire designed to measure ability to develop, implement, and maintain planned behavior to achieve one’s goals. Examinees respond to each item using the 5-point Likert scale: 1 = *Strongly disagree*, 2 = *Disagree*, 3 = *Uncertain or Unsure*, 4 = *Agree*, 5 = *Strongly Agree*.

#### Self Control Scale (SCS)

The SCS [41] is a 36-item questionnaire designed to measure self-control, that is, the self-control over thoughts, emotional control, impulse control, performance regulation, and habit breaking. Examinees rated each statement on a 5-point scale ranging from 1 = N*ot at all like me* to 5 = V*ery much like me*.

### Procedure

Participants were tested in small groups of 1 to 10 participants, seated widely separated in a small classroom, in a single session lasting approximately 2 hours, by one of eight trained research assistants. Table 1 indicates the order of the tasks and the approximate time required for completing each task. First, participants provided written informed consent and basic demographic information (age, gender, whether their first language was English, handedness, major). Second, they completed a set of timed tasks (DS Backward, DS Coding). Lastly, they completed the remaining tasks at their own pace, placing each completed page on the desk next to them (this prevented them from going back and circling cues they may not have responded to at the right time).

## Results

### Data screening

Following the recommendations for outlier detection [42,43], the data were screened for univariate outliers, defined as scores falling more than 3.5 MADe distances from the median (MAD = Median Absolute Deviation and MADe = 0.6745*MAD). A fewer than 0.1% of all values on any of the variables were identified as outliers. The influence of outliers was reduced by replacing them with corresponding outlier caps (i.e., a value 3.5*MADe from a median, either below or above, as appropriate).

### Continuous versus binary measures of ProM

Table 2 shows the means, standard deviations, and reliabilities for individual ProM assessments as well as for the composite ProM measures (i.e., averages of the two assessments). The data are shown separately for men, women, and for all participants combined. As expected, the correlation between the two binary measures of ProM was small, *r* = .11. In contrast, the correlation between the two continuous measures of ProM was much larger, *r* = .50. In turn, the Cronbach’s *α* of the binary composite was much smaller than that of the continuous composite, .19 versus .64. In comparison, the Cronbach’s *α* of the RetM tests–VLT/C20, calculated over three study-test trials, was .87. Our results confirm a well-known mathematical fact that binary measures are unable to measure individual differences in abilities with any degree of precision, are unreliable, and inefficient.

**Table 2:** Descriptive statistics and reliabilities.

	Men		Women		All		
	*M*	*SD*	*M*	*SD*	*M*	*SD*	α
ProM/B	0.21	0.32	0.21	0.30	0.21	0.30	0.19
ProM/C	5.78	1.87	5.91	1.67	5.88	1.71	0.64
VLT/C20	12.33	2.55	13.21	2.24	13.02	2.33	0.87
Words/A40	0.61	0.17	0.61	0.16	0.61	0.17	0.83
SILS/R	0.77	0.14	0.75	0.14	0.75	0.14	0.79
DS Coding	74.78	15.17	78.25	14.27	77.53	14.60	
DS Backward	5.69	1.41	5.44	1.40	5.49	1.41	
IPIP100 Agreeableness	3.76	0.53	4.00	0.48	3.96	0.50	0.86
IPIP100 Conscientiousness	3.40	0.56	3.46	0.59	3.45	0.58	0.89
IPIP100 Emotional Stability	3.26	0.72	2.90	0.71	2.98	0.72	0.92
IPIP100 Extroversion	3.22	0.68	3.19	0.70	3.20	0.69	0.92
IPIP100 Intellect (Openness)	3.66	0.48	3.49	0.52	3.53	0.51	0.86
SCL90R GSI	1.00	0.66	1.12	0.67	1.09	0.67	0.98
SCL90R PST	45.06	20.33	47.59	19.04	47.05	19.38	
SCL90R PSDI	1.86	0.54	1.97	0.55	1.95	0.55	0.98
SCL90R SOM	0.86	0.70	1.04	0.77	1.00	0.76	0.87
SCL90R O-C	1.56	0.84	1.70	0.87	1.67	0.86	0.87
SCL90R I-S	1.10	0.82	1.31	0.88	1.26	0.87	0.87
SCL90R DEP	1.15	0.82	1.41	0.89	1.36	0.88	0.91
SCL90R ANX	0.86	0.74	1.00	0.80	0.97	0.79	0.87
SCL90R HOS	0.95	0.79	0.86	0.77	0.87	0.77	0.82
SCL90R PHOB	0.35	0.60	0.52	0.67	0.48	0.66	0.81
SCL90R PAR	1.18	0.92	1.08	0.85	1.10	0.87	0.79
SCL90R PSY	0.77	0.76	0.70	0.69	0.71	0.70	0.83

*Note*. ProM/B = ProM binary measure, ProM/C = ProM continuous measure; VLT/C20 = Verbal Learning Test Categorized 20, Words/A40 = Verbal knowledge test, SILS/R = Shippley's Institute for Living Scale Reasoning Test, DS Coding = Digit Symbol Coding, IPIP100 = International Personality Item Pool 100 Item Big Five, SCL90R = Symptoms Checklist 90 Revised, GSI = Global Severity Index, PST = Positive Symptom Total, PSDI = Positive Symptom Distress Index, SOM = Somatization, O-C = Obsessive-Compulsive, I-S = Interpersonal Sensitivity, DEP = Depression, ANX = Anxiety, HOS = Hostility, PHOB = Phobic Anxiety, PAR = Paranoid Ideation, PSY = Psychoticism.

Accordingly, the binary measures of ProM are reported for comparison purposes only and to highlight their inferior psychometric properties. The continuous measures of ProM are used for all substantive analyses to address objectives of this study.

**Fig 1:**
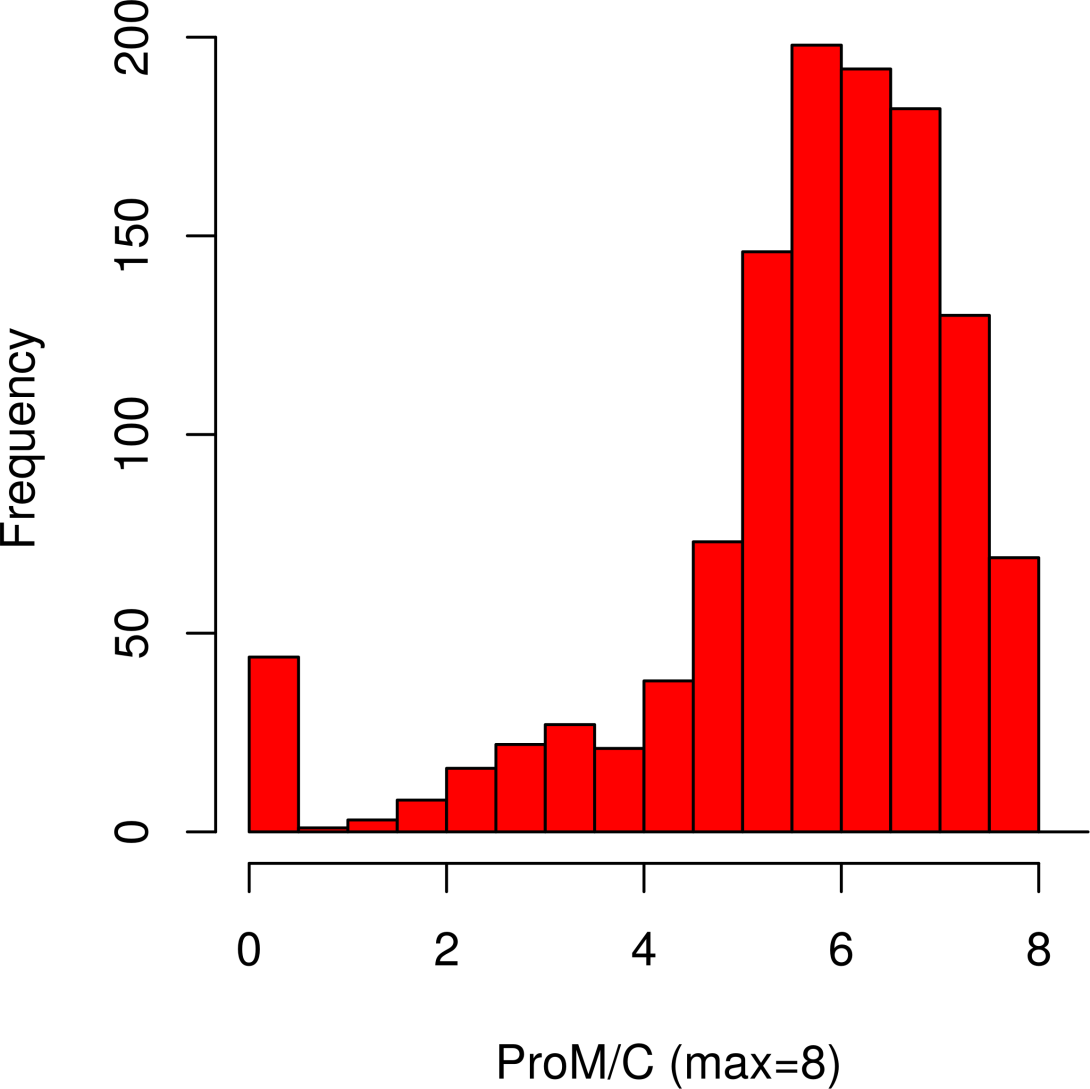
Distribution of ProM/C (continuous measure of ProM). The distribution of ProM/C was relatively normal with a negative skew. Only a small number of participants scored at the floor or at the ceiling.

Fig 1 shows the distribution of the ProM/C, a continuous measure of ProM. The distribution is relatively close to normal, with a negative skew (-1.72), and with a relatively small number of participants scoring either at the ceiling (6.0%) or at the floor (3.7%).

### ProM, RetM, intelligence, working memory, and processing speed

Table 2 also shows the means, standard deviations, and reliabilities for all other measures. Table 3 shows a partial correlation matrix showing correlations among ProM, RetM, intelligence, working memory, and processing speed measures.

**Table 3:** Correlation matrix for all participants.

	1	2	3	4	5	6	7	8
1. Sex (F = 1, M = 0)		0.01	0.03	**0.15**	-0.02	-0.07	0.10	-0.07
2. ProM/B	0.01		**0.58**	**0.11**	**0.19**	**0.17**	**0.06**	**0.07**
3. ProM/C	0.03	**0.58**		**0.22**	**0.28**	**0.33**	**0.15**	**0.11**
4. VLT/C20	**0.15**	**0.11**	**0.22**		**0.23**	**0.33**	**0.24**	**0.22**
5. Words/A40	-0.02	**0.19**	**0.28**	**0.23**		**0.36**	**0.12**	**0.19**
6. SILS/R	**-0.07**	**0.17**	**0.33**	**0.33**	**0.36**		**0.33**	**0.37**
7. DS Coding	**0.10**	**0.06**	**0.15**	**0.24**	**0.12**	**0.33**		**0.13**
8. DS Backward	**-0.07**	**0.07**	**0.11**	**0.22**	**0.19**	**0.37**	**0.13**	
9. IPIP100 Agreeableness	**0.19**	0.03	0.02	0.05	**0.07**	-0.04	-0.02	0.04
10. IPIP100 Conscientiousness	0.04	0.00	0.00	0.04	0.02	**-0.06**	0.05	0.01
11. IPIP100 Emotional Stability	**-0.20**	0.04	0.01	0.01	**0.10**	**0.10**	**0.06**	**0.10**
12. IPIP100 Extroversion	-0.01	-0.03	-0.04	0.02	-0.04	-0.05	0.01	**0.07**
13. IPIP100 Intellect (Openness)	**-0.13**	0.05	**0.08**	**0.09**	**0.31**	**0.07**	0.04	**0.08**
14. SCL90R GSI	**0.07**	-0.05	**-0.07**	**-0.07**	**-0.17**	**-0.09**	**-0.09**	**-0.10**
15. SCL90R PST	0.05	-0.01	**-0.06**	-0.05	**-0.17**	**-0.06**	**-0.10**	**-0.10**
16. SCL90R PSDI	**0.08**	**-0.09**	**-0.08**	**-0.09**	**-0.15**	**-0.12**	**-0.07**	**-0.11**
17. SCL90R SOM	**0.10**	**-0.06**	-0.05	-0.03	**-0.14**	**-0.07**	**-0.06**	**-0.08**
18. SCL90R O-C	**0.06**	-0.03	**-0.06**	**-0.12**	**-0.15**	**-0.08**	**-0.11**	**-0.12**
19. SCL90R I-S	**0.09**	-0.02	-0.05	-0.04	**-0.10**	**-0.06**	**-0.07**	**-0.10**
20. SCL90R DEP	**0.12**	-0.05	**-0.06**	-0.04	**-0.11**	**-0.08**	**-0.06**	**-0.10**
21. SCL90R ANX	**0.07**	-0.05	**-0.07**	-0.04	**-0.15**	**-0.08**	**-0.08**	**-0.10**
22. SCL90R HOS	-0.05	**-0.09**	**-0.10**	**-0.08**	**-0.17**	**-0.09**	**-0.07**	-0.04
23. SCL90R PHOB	**0.10**	-0.05	**-0.06**	-0.02	**-0.19**	**-0.11**	**-0.06**	**-0.08**
24. SCL90R PAR	-0.04	-0.05	**-0.10**	**-0.12**	**-0.20**	**-0.11**	**-0.08**	**-0.09**
25. SCL90R PSY	-0.04	-0.02	**-0.07**	**-0.10**	**-0.20**	**-0.07**	**-0.07**	**-0.10**

*Note*. *N* = 1170. Bold print *p* < .05. ProM/B = ProM binary measure, ProM/C = ProM continuous measure, Words/A40 = Verbal knowledge test, VLT/C20 = Verbal Learning Test Categorized 20, SILS/R = Shippley's Institute for Living Scale Reasoning Test, DS Coding = Digit Symbol Coding, IPIP100 = International Personality Item Pool 100 Item Big Five, SCL90R = Symptoms Checklist 90 Revised, GSI = Global Severity Index, PST = Positive Symptom Total, PSDI = Positive Symptom Distress Index, SOM = Somatization, O-C = Obsessive-Compulsive, I-S = Interpersonal Sensitivity, DEP = Depression, ANX = Anxiety, HOS = Hostility, PHOB = Phobic Anxiety, PAR = Paranoid Ideation, PSY = Psychoticism.

**Fig 2:**
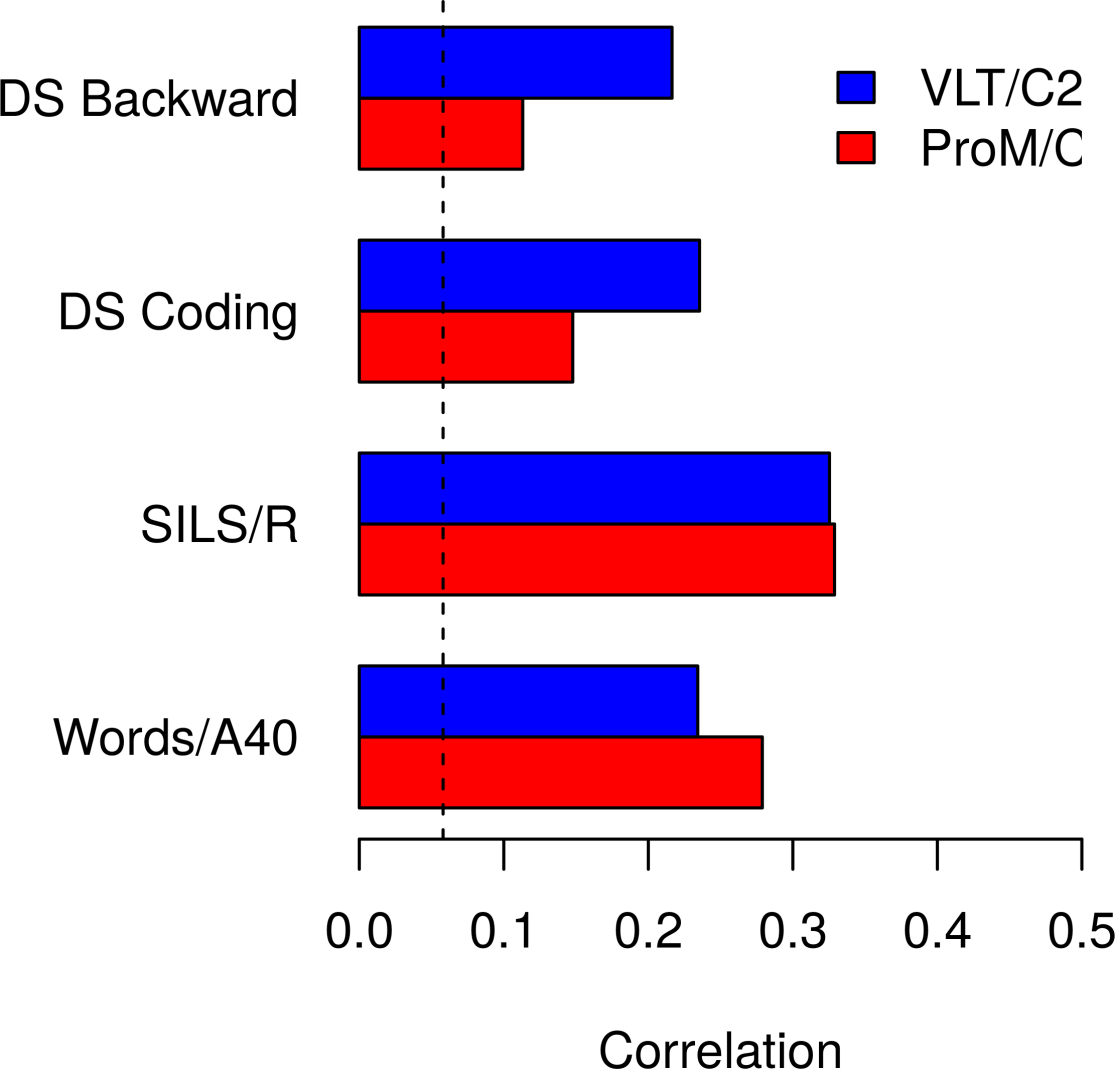
Correlations between ProM and RetM with intelligence, processing speed, and working memory. ProM versus RetM correlations with crystallized and fluid intelligence were comparable. In contrast, correlations with processing speed and working memory were smaller for ProM versus RetM.

Fig 2 contrasts the correlations between ProM versus RetM and Words/A40, SILS/Reasoning, DS Backward and DS Coding. First, for intelligence measures, SILS/R and Words/A40, the correlations with ProM and RetM were comparable (Words/A40: *z* = 1.434, *p* = .076; SILS/R: *z* = 0, *p* = .500). Second, for both processing speed and working memory measures, the correlations were stronger with RetM than with ProM (DS Coding: *z* = 2.531, *p* = .006; DS Backwards: *z* = 3.071, *p* = .001).

### ProM, RetM, personality and symptoms of psychopathology

**Fig 3:**
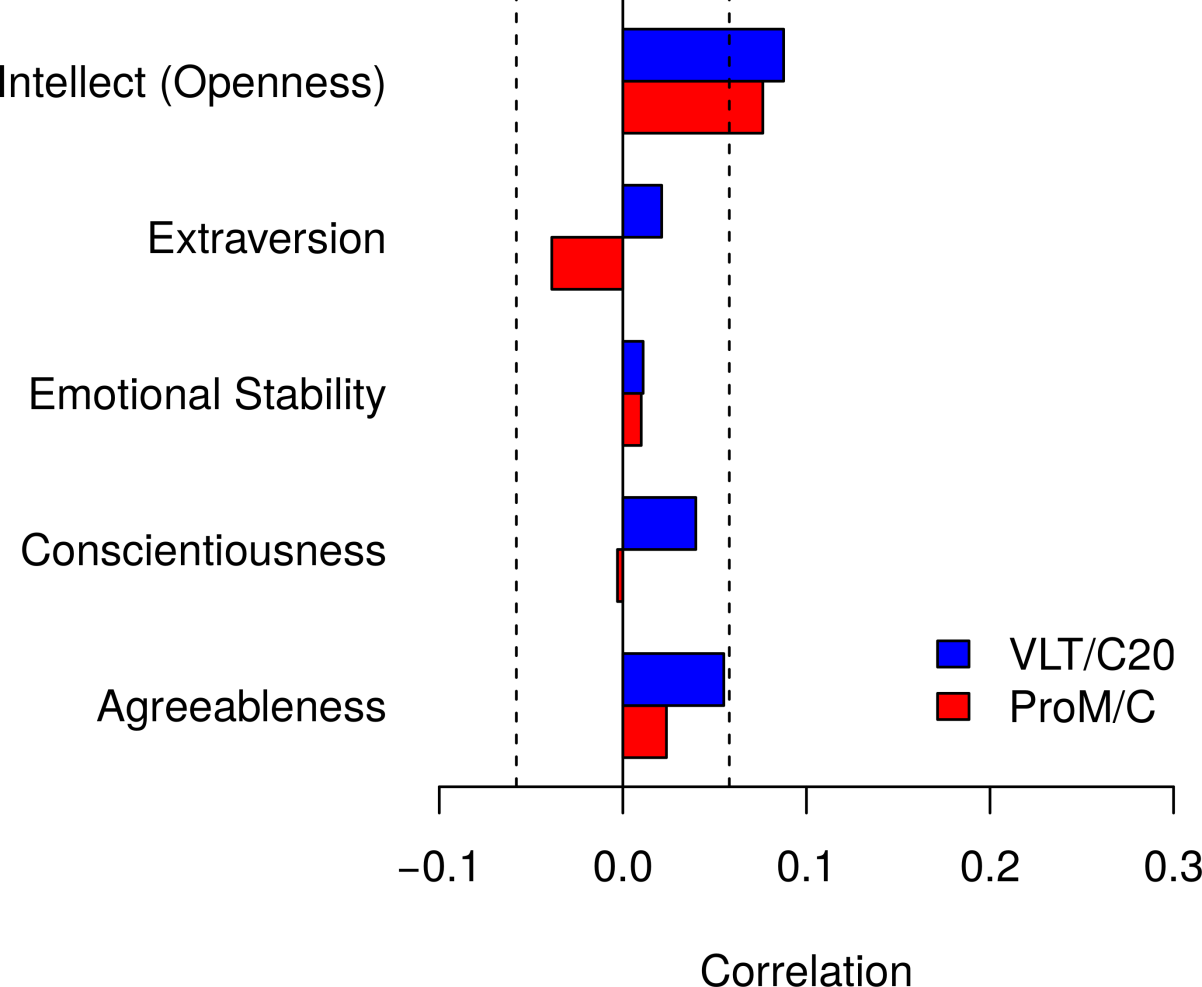
Correlations between ProM and personality. Both ProM and RetM correlated only with Openness and the two correlations were comparable. The dashed lines indicate *p* = .05 criterion.

Table 3 also shows correlations between ProM versus RetM and personality and symptoms of psychopathology. Fig 3 highlights that out of the Big Five, as measured by IPIP100, only Intellect/Imagination (Openness) correlated with ProM with a correlation of, *r* = .09. Fig 3 also highlights the same pattern of correlations between the Big Five and ProM for RetM.

**Fig 4:**
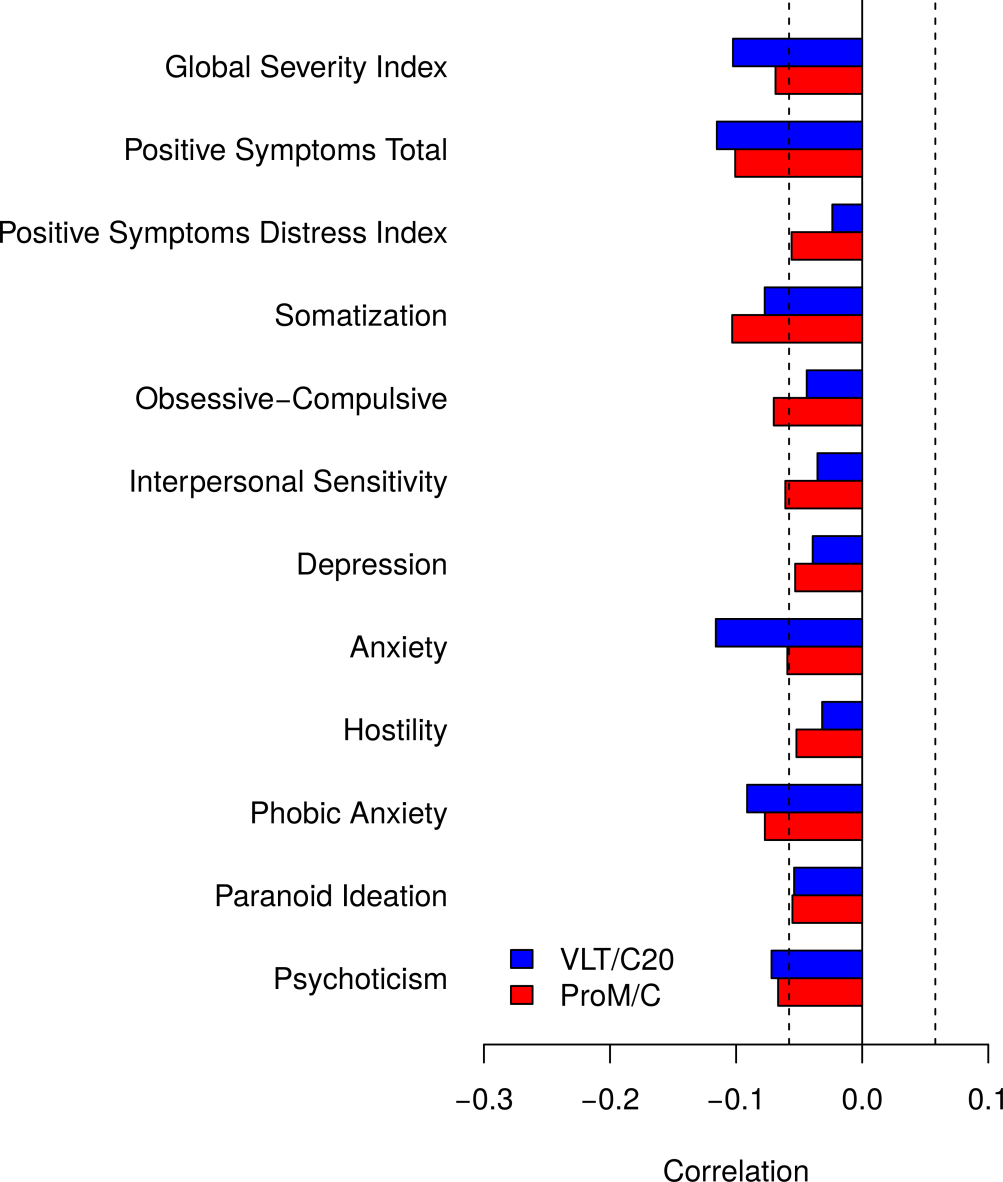
Correlations between ProM and symptoms of psychopathology. In general, both ProM and RetM showed very small negative correlations with symptoms of psychopathology. The dashed lines indicate *p* = .05 criterion.

Fig 4 shows that, in general, symptoms of psychopathology were negatively correlated with ProM but that such correlations were weak, < .10, and only Hostility and Paranoia reached .10. Similar weak correlations were found between symptoms of psychopathology and RetM.

### Sex differences in ProM

Table 3 includes correlations between sex and measures of ProM as well as between sex and all other measures. The correlation between sex and ProM was near zero, *r* = .03, and not statistically significant even though there was a small significant correlation between sex and RetM, *r* = .15.

Given the large sample, Table 4 and Table 5 show the partial correlation matrix among ProM, RetM, and other measures for men and women, respectively. The correlations between ProM and other measures were similar for men and women.

**Table 4:** Correlation matrix for men.

	1	2	3	4	5	6	7
1. ProM/B		**0.57**	0.04	**0.14**	**0.17**	**0.07**	0.04
2. ProM/C	**0.57**		**0.18**	**0.29**	**0.34**	**0.15**	**0.16**
3. VLT/C20	0.04	**0.18**		**0.20**	**0.31**	**0.27**	**0.26**
4. Words/A40	**0.14**	**0.29**	**0.20**		**0.31**	**0.16**	**0.16**
5. SILS/R	**0.17**	**0.34**	**0.31**	**0.31**		**0.42**	**0.39**
6. DS Coding	0.07	**0.15**	**0.27**	**0.16**	**0.42**		**0.21**
7. DS Backward	0.04	**0.16**	**0.26**	**0.16**	**0.39**	**0.21**	
8. IPIP100 Agreeableness	-0.03	-0.08	0.00	0.02	-0.08	-0.06	0.06
9. IPIP100 Conscientiousness	-0.06	-0.02	-0.03	-0.01	**-0.14**	0.01	-0.10
10. IPIP100 Emotional Stability	**0.15**	0.09	0.12	**0.20**	0.06	0.11	0.10
11. IPIP100 Extroversion	-0.06	**-0.14**	-0.01	**-0.13**	-0.08	-0.02	0.04
12. IPIP100 Intellect (Openness)	-0.02	-0.03	**0.14**	**0.23**	0.03	0.02	0.02
13. SCL90R GSI	-0.09	-0.08	**-0.14**	**-0.17**	-0.02	-0.03	**-0.13**
14. SCL90R PST	-0.11	-0.07	-0.12	**-0.17**	0.04	-0.04	-0.12
15. SCL90R PSDI	-0.09	**-0.14**	**-0.19**	**-0.13**	**-0.14**	-0.05	**-0.16**
16. SCL90R SOM	-0.09	-0.02	-0.01	-0.10	0.06	0.05	0.00
17. SCL90R O-C	-0.11	-0.06	**-0.24**	-0.12	-0.05	-0.10	**-0.14**
18. SCL90R I-S	-0.07	-0.05	-0.12	**-0.13**	0.01	-0.03	**-0.18**
19. SCL90R DEP	-0.09	-0.08	-0.11	**-0.13**	-0.03	-0.02	**-0.14**
20. SCL90R ANX	-0.05	-0.06	-0.12	**-0.14**	0.00	0.01	-0.10
21. SCL90R HOS	-0.12	**-0.14**	-0.12	**-0.24**	-0.11	**-0.13**	**-0.13**
22. SCL90R PHOB	-0.03	-0.02	-0.10	**-0.13**	-0.04	-0.05	-0.12
23. SCL90R PAR	-0.11	**-0.13**	**-0.17**	**-0.16**	-0.04	-0.02	-0.11
24. SCL90R PSY	-0.01	-0.05	**-0.13**	**-0.20**	-0.02	0.00	**-0.14**

*Note*. *N* = 232. Bold print *p* < .05. ProM/B = ProM binary measure, ProM/C = ProM continuous measure, Words/A40 = Verbal knowledge test, VLT/C20 = Verbal Learning Test Categorized 20, SILS/R = Shippley's Institute for Living Scale Reasoning Test, DS Coding = Digit Symbol Coding, IPIP100 = International Personality Item Pool 100 Item Big Five, SCL90R = Symptoms Checklist 90 Revised, GSI = Global Severity Index, PST = Positive Symptom Total, PSDI = Positive Symptom Distress Index, SOM = Somatization, O-C = Obsessive-Compulsive, I-S = Interpersonal Sensitivity, DEP = Depression, ANX = Anxiety, HOS = Hostility, PHOB = Phobic Anxiety, PAR = Paranoid Ideation, PSY = Psychoticism.

**Table 5:** Correlation matrix for women.

	1	2	3	4	5	6	7
1. ProM/B		**0.58**	**0.13**	**0.20**	**0.18**	**0.06**	**0.09**
2. ProM/C	**0.58**		**0.23**	**0.28**	**0.33**	**0.15**	**0.11**
3. VLT/C20	**0.13**	**0.23**		**0.25**	**0.35**	**0.20**	**0.22**
4. Words/A40	**0.20**	**0.28**	**0.25**		**0.38**	**0.11**	**0.21**
5. SILS/R	**0.18**	**0.33**	**0.35**	**0.38**		**0.32**	**0.36**
6. DS Coding	0.06	**0.15**	**0.20**	**0.11**	**0.32**		**0.12**
7. DS Backward	**0.09**	**0.11**	**0.22**	**0.21**	**0.36**	**0.12**	
8. IPIP100 Agreeableness	0.04	0.05	0.03	**0.09**	-0.01	-0.03	0.05
9. IPIP100 Conscientiousness	0.01	0.00	0.05	0.02	-0.04	0.06	0.05
10. IPIP100 Emotional Stability	0.01	0.00	0.02	**0.08**	**0.09**	**0.07**	**0.08**
11. IPIP100 Extroversion	-0.02	-0.01	0.03	-0.02	-0.04	0.02	**0.07**
12. IPIP100 Intellect (Openness)	0.06	**0.11**	0.10	**0.33**	**0.07**	0.06	**0.08**
13. SCL90R GSI	-0.03	**-0.07**	**-0.07**	**-0.17**	**-0.09**	**-0.12**	**-0.09**
14. SCL90R PST	0.02	-0.05	-0.05	**-0.16**	**-0.08**	**-0.12**	**-0.10**
15. SCL90R PSDI	**-0.09**	**-0.07**	**-0.08**	**-0.15**	**-0.11**	**-0.10**	**-0.10**
16. SCL90R SOM	-0.05	-0.06	-0.06	**-0.15**	**-0.09**	**-0.10**	**-0.08**
17. SCL90R O-C	0.00	-0.06	**-0.10**	**-0.16**	**-0.07**	**-0.13**	**-0.11**
18. SCL90R I-S	0.00	-0.05	-0.04	**-0.09**	-0.06	**-0.10**	**-0.07**
19. SCL90R DEP	-0.04	-0.06	-0.04	**-0.11**	**-0.08**	**-0.09**	**-0.08**
20. SCL90R ANX	-0.05	**-0.08**	-0.04	**-0.15**	**-0.09**	**-0.11**	**-0.10**
21. SCL90R HOS	**-0.08**	**-0.09**	-0.06	**-0.16**	**-0.08**	-0.04	-0.02
22. SCL90R PHOB	-0.05	**-0.07**	-0.02	**-0.19**	**-0.11**	**-0.08**	-0.06
23. SCL90R PAR	-0.02	**-0.09**	**-0.09**	**-0.21**	**-0.13**	**-0.10**	**-0.09**
24. SCL90R PSY	-0.02	**-0.07**	**-0.08**	**-0.20**	**-0.09**	**-0.09**	**-0.10**

*Note*. *N* = 923. Bold print *p* < .05. ProM/B = ProM binary measure, ProM/C = ProM continuous measure, Words/A40 = Verbal knowledge test , VLT/C20 = Verbal Learning Test Categorized 20, SILS/R = Shippley's Institute for Living Scale Reasoning Test, DS Coding = Digit Symbol Coding, IPIP100 = International Personality Item Pool 100 Item Big Five, SCL90R = Symptoms Checklist 90 Revised, GSI = Global Severity Index, PST = Positive Symptom Total, PSDI = Positive Symptom Distress Index, SOM = Somatization, O-C = Obsessive-Compulsive, I-S = Interpersonal Sensitivity, DEP = Depression, ANX = Anxiety, HOS = Hostility, PHOB = Phobic Anxiety, PAR = Paranoid Ideation, PSY = Psychoticism.

### Regression analyses

We conducted several hierarchical regression analyses to predict performance on ProM versus RetM measures using cognitive ability, personality, and symptoms of psychopathology. In the first set of analyses, the cognitive variables measuring processing speed, working memory and crystalized and fluid intelligence were entered first, and personality and symptoms of psychopathology measures were added on subsequent steps. These analyses allows us to see how much additional variance personality and psychopathology measures explain above and beyond cognitive measures. In the second set of analyses, suggested by the reviewers, the personality and symptoms of psychopathology measures were entered first and cognitive measures on subsequent steps. This second set of analyses allows us to see how much additional variance cognitive measures explain above and beyond personality and symptoms of psychopathology measures.

Table 6 shows the results of the first set of the hierarchical regression analyses. On the first step, we entered DS Coding, a measure of processing speed. On the second step, we added DS Backward, a measure of working memory. On the third step, we added the two measures of intelligence, Words/A40 and SILS/R. On the fourth step, for predicting ProM only, we added VLT/C20, a measure of RetM. On the fifth step, we added personality measures, and, on the fifth alternative step, we added measures of symptoms of psychopathology. When predicting ProM, on each of the first four steps, the added variables significantly improved prediction but, when intelligence measures were added into the model, DS Coding and DS Backward coefficients were no longer statistically significant. In total, cognitive ability variables explained 14.9% of variability in ProM. On the fifth step, neither personality nor symptoms of psychopathology measures improved prediction beyond cognitive ability.

**Table 6:** Summary of hierarchical regression analyses predicting ProM versus RetM from cognitive ability, personality, and symptoms of psychopathology.

	ProM				RetM			
	*β*	*R2*	*ΔR2*	*F of ΔR2*	*β*	*R2*	*ΔR2*	*F of ΔR2*
**Step 1**		.022	.022	**26.05**		.055	.055	**68.55**
DS Coding	**.147**				**.235**			
**Step 2**		.031	.009	**12.13**		.090	.035	**47.96**
DS Coding	**.135**				**.210**			
DS Backward	**.095**				**.189**			
**Step 3**		.140	.109	**74.21**		.149	.059	**40.02**
DS Coding	.043				**.142**			
DS Backward	-.021				**.102**			
Words/A40	**.185**				**.127**			
SILS/R	**.255**				**.194**			
**Step 4**		.149	.009	**12.22**				
DS Coding	.029							
DS Backward	-.032							
Words/A40	**.172**							
SILS/R	**.235**							
VLT/C20	**.103**							
**Step 5**		.150	.001	0.50		.156	.007	2.02
DS Coding	.031				**.140**			
DS Backward	-.029				**.100**			
Words/A40	**.170**				**.127**			
SILS/R	**.238**				**.208**			
VLT/C20	**.100**							
Agreeableness	.028				.043			
Conscientiousness	.007				.046			
Emotional Stability	-.029				**-.066**			
Extroversion	-.025				.026			
Intellect (Openness)	.003				-.001			
**Step 5**		.153	.003	0.71		.173	.024	**3.85**
DS Coding	.029				**.130**			
DS Backward	-.029				**.099**			
Words/A40	**.172**				**.104**			
SILS/R	**.231**				**.205**			
VLT/C20	**.103**							
SCL90R SOM	.018				.016			
SCL90R O-C	.052				**-.176**			
SCL90R I-S	-.021				.082			
SCL90R DEP	-.011				**.128**			
SCL90R ANX	-.050				.073			
SCL90R HOS	-.057				-.024			
SCL90R PHOB	.035				.075			
SCL90R PAR	-.037				-.074			
SCL90R PSY	.050				**-.123**			

*Note*. *N* = 1170. Bold print *p* < .05. DS Coding = Digit Symbol Coding, DS Backward = Digit Span Backwards, Words/A40 = Verbal knowledge test, SILS/R = Shippley's Institute for Living Scale Reasoning Test, VLT/C20 = Verbal Learning Test Categorized 20; SCL90R = Symptoms Checklist 90 Revised, GSI = Global Severity Index, PST = Positive Symptom Total, PSDI = Positive Symptom Distress Index, SOM = Somatization, O-C = Obsessive-Compulsive, I-S = Interpersonal Sensitivity, DEP = Depression, ANX = Anxiety, HOS = Hostility, PHOB = Phobic Anxiety, PAR = Paranoid Ideation, PSY = Psychoticism.

When predicting RetM, on each of the first three steps, the added variables significantly improved prediction, with DS Coding and DS Backward's contributions being stronger than for ProM. Moreover, when intelligence measures were added into the model, DS Coding and DS Backward coefficients remained statistically significant. In total, cognitive ability variables also explained 14.9% of variability in RetM. Similar to ProM, personality did not improve the prediction of RetM beyond cognitive ability. However, symptoms of psychopathology improved the prediction of RetM slightly, (1.7% increase in *R2*).

Table 7 shows the results of the second set of the hierarchical regression analyses. On the first step, we entered personality variables. On the second step, we added symptoms of psychopathology. On the third step, we added DS Coding, a measure of processing speed. On the fourth step, we added DS Bakward, a measure of working memory. On the fifth step, we added two measures of intelligence, Words/A40 and SILS/R. And on the sixth, final step, we added VLT/C20, a measure of RetM.

**Table 7:** Summary of hierarchical regression analyses predicting ProM versus RetM from personality, symptoms of psychopathology, and cognitive ability.

	ProM				RetM			
	*β*	*R2*	*ΔR2*	*F of ΔR2*	*β*	*R2*	*ΔR2*	*F of ΔR2*
**Step 1**		.012	.012	**2.78**		.009	.009	2.133
Agreeableness	.027				.034			
Conscientiousness	-.028				.016			
Emotional Stability	.021				-.068			
Extroversion	**-.085**				-.018			
Intellect (Openness)	.100				.080			
**Step 2**		.033	.021	**3.16**		.048	.039	**6.111**
Agreeableness	.009				.016			
Conscientiousness	-.041				-.010			
Emotional Stability	-.079				-.041			
Extroversion	**-.088**				.010			
Intellect (Openness)	**.119**				**.078**			
SCL90R SOM	.026				.014			
SCL90R O-C	.030				**-.200**			
SCL90R I-S	.023				**.145**			
SCL90R DEP	-.036				.122			
SCL90R ANX	-.043				.061			
SCL90R HOS	**-.093**				-.043			
SCL90R PHOB	-.020				.044			
SCL90R PAR	**-.112**				**-.138**			
SCL90R PSY	-.031				**-.127**			
**Step 3**		.052	.019	**26.28**		.096	0.48	**66.742**
Agreeableness	.016				.026			
Conscientiousness	-.045				-.016			
Emotional Stability	**-.080**				-.043			
Extroversion	**-.087**				.012			
Intellect (Openness)	**.114**				**.071**			
SCL90R SOM	.023				.010			
SCL90R O-C	.048				**-.171**			
SCL90R I-S	.022				**.143**			
SCL90R DEP	-.048				.103			
SCL90R ANX	-.042				.063			
SCL90R HOS	**-.089**				-.038			
SCL90R PHOB	-.018				.046			
SCL90R PAR	**-.107**				**-.130**			
SCL90R PSY	.032				**-.125**			
DS Coding	**.140**				**.220**			
**Step 4**		.060	.008	**11.17**		.127	.031	**43.82**
greeableness	.013				.022			
Conscientiousness	-.040				-.062			
Emotional Stability	**-.086**				-.054			
Extroversion	**-.088**				.011			
Intellect (Openness)	**.109**				**.061**			
SCL90R SOM	.025				.014			
SCL90R O-C	.054				**-.160**			
SCL90R I-S	.020				**.140**			
SCL90R DEP	-.048				.103			
SCL90R ANX	-.039				.069			
SCL90R HOS	**-.098**				-.054			
SCL90R PHOB	-.020				.042			
SCL90R PAR	**-.106**				**-.128**			
SCL90R PSY	.039				**-.114**			
DS Coding	**.123**				**.198**			
DS Backward	**.093**				**.180**			
**Step 5**		.150	.090	**61.80**		.180	.053	**37.026**
Agreeableness	.024				.030			
Conscientiousness	-.001				-.024			
Emotional Stability	**-.102**				-.068			
Extroversion	-.026				.054			
Intellect (Openness)	.018				.028			
SCL90R SOM	.030				.016			
SCL90R O-C	.030				**-.179**			
SCL90R I-S	-.043				.095			
SCL90R DEP	-.038				.115			
SCL90R ANX	-.060				.052			
SCL90R HOS	-.076				-.038			
SCL90R PHOB	.034				**.082**			
SCL90R PAR	-.035				-.077			
SCL90R PSY	.056				**-.105**			
DS Coding	.042				**.128**			
DS Backward	-.015				**.095**			
Words/A40	**.175**				**.105**			
SILS/R	**.256**				**.217**			
**Step 6**		.159	.009	**11.40**				
Agreeableness	.021							
Conscientiousness	-.002							
Emotional Stability	**-.095**							
Extroversion	-.032							
Intellect (Openness)	.018							
SCL90R SOM	.028							
SCL90R O-C	.048							
SCL90R I-S	-.053							
SCL90R DEP	-.049							
SCL90R ANX	-.065							
SCL90R HOS	-.072							
SCL90R PHOB	.026							
SCL90R PAR	-.028							
SCL90R PSY	.067							
DS Coding	.030							
DS Backward	-.026							
Words/A40	**.164**							
SILS/R	**.234**							
VLT/C20	**.100**							

*Note*. *N* = 1170. Bold print *p* < .05. DS Coding = Digit Symbol Coding, DS Backward = Digit Span Backwards, Words/A40 = Verbal knowledge test, SILS/R = Shippley's Institute for Living Scale Reasoning Test, VLT/C20 = Verbal Learning Test Categorized 20; SCL90R = Symptoms Checklist 90 Revised, GSI = Global Severity Index, PST = Positive Symptom Total, PSDI = Positive Symptom Distress Index, SOM = Somatization, O-C = Obsessive-Compulsive, I-S = Interpersonal Sensitivity, DEP = Depression, ANX = Anxiety, HOS = Hostility, PHOB = Phobic Anxiety, PAR = Paranoid Ideation, PSY = Psychoticism.

When predicting ProM, the added variables significantly improved prediction at each step. The personality and symptoms of psychopathology explained only 3.3% of variance. In contrast, cognitive variables explained additional 12.6% of variance above and beyond the personality and symptoms of psychopathology variables. Similarly, when predicting RetM, the added variables significantly improved prediction at each step. The personality and symptoms of psychopathology explained 4.8% of variance in RetM. The cognitive variables explained additional 13.2% of variance above and beyond the personality and symptoms of psychopathology variables.

## Discussion

The current study resulted in the following key findings. First, continuous measures of episodic event-cued ProM were more reliable than binary measures derived from the same ProM task. The reliability of a composite continuous measure of ProM was respectable, .64. Accordingly, as expected [2,44], the composite of two continuous versus binary measures of ProM showed larger correlations with other measures, including measures of cognitive ability, personality, and symptoms of psychopathology. Second, episodic event-cued ProM was associated with measures of processing speed, working memory, crystallized and fluid intelligence, as well as RetM. Moreover, processing speed, working memory, and intelligence explained comparable proportions of variance in ProM and RetM. Third, personality factors–Big Five measured by IPIP100 –did not improve the prediction of neither ProM nor RetM beyond variance predicted by cognitive ability. Fourth, symptoms of psychopathology did not improve the prediction of ProM although they slightly improved the prediction of RetM. When personality and symptoms of psychopathology were entered first in the hierarchical regression analyses, they explained only 3.3% and 4.8% variance in ProM and RetM, respectively. Cognitive measures explained additional 12.6% and 15.2% of variance in ProM and RetM, respectively, above and beyond the variance explained by personality and symptoms of psychopathology. Finally, participants' sex was not associated with ProM but showed small correlations with RetM.

The use of unreliable binary measures in the investigations of ProM has two direct, undesirable consequences. First, because unreliable measures measure primarily error variation rather than ability of interest, researchers must use large sample sizes to increase statistical power and to compensate for the low reliability of binary measures. As a corollary, studies with small samples are unlikely to find any statistically significant correlations between binary ProM measures and other abilities and constructs. Since most of the previous studies investigating relationships between ProM and other individual differences used binary measures of ProM and small samples, it is not surprising that they often reported no correlations between ProM and intelligence, personality, and symptoms of psychopathology.

Second, the use of unreliable binary measures of ProM makes it nearly impossible to compare magnitudes of correlations between ProM and other constructs and variables because of large differences in reliability of the measures. For example, for theoretical reasons [45], researchers have been interested in determining whether ProM versus RetM show larger age-related declines across the adult life span. However, when there are large differences in the reliabilities of ProM and RetM measures used in a particular study, researchers are more likely to find that age declines on ProM measures are smaller than age declines on RetM measures simply because of the differences in the reliabilities of the two measures rather than the differences in the size of the true age declines themselves [2].

We found substantial correlations between episodic event-cued ProM and cognitive ability measures. Both crystallized/verbal intelligence and fluid/performance intelligence measures correlated with episodic ProM and these correlations were as large as with RetM. Processing speed and working memory correlated somewhat weaker with ProM than with RetM. This latter finding is consistent with the views that processing speed and working memory are more important for both encoding and retrieval in RetM, less important for encoding of the ProM cue and plan, and even less important for retrieval in ProM. For example, in our study, participants only had a limited time to encode 20 to-be-remembered words for the RetM test, whereas they had unlimited time to encode only one ProM cue. However, although cognitive abilities explained a similar proportion of variance in ProM and RetM measures in our study, it is possible and even likely that cognitive abilities would explain a larger proportion of variability in ProM than in RetM if reliabilities of ProM and RetM measures were equal.

Our examination of the relationships between episodic ProM and personality showed that out of the Big Five, only the Openness (measured by IPIP Intellect) was correlated with episodic ProM, and that this correlation was very small, *r* = .08. This finding is consistent with both (a) our meta-analysis of previously published studies suggesting that any correlations between personality and ProM were small (*r* ~ .10) and (b) our large sample study (*N* = 283) showing no statistically significant correlations between episodic ProM and the Big Five (measured by two different personality inventories) even though episodic ProM correlated .09 with NEO Openness and .04 with IPIP Openness [17]. Although it may seem surprising that, for example, Conscientiousness was not correlated with episodic ProM, Conscientiousness may not influence performance in controlled laboratory conditions [17,30]. Personality factors such as Conscientiousness may be more influential if episodic ProM was assessed in natural conditions and/or when completion of an episodic ProM task is of greater social importance to participants. Personality factors may be more influential if ProM was assessed in natural conditions, the ProM cue was time rather than an event, and the ProM task was socially important [17,46]. In our earlier study, we [17] found that personality factors affect subdomains of ProM (i.e., episodic ProM versus vigilance/monitoring) differently and it would not be surprising if the relationship between personality factors and various ProM subdomains also differed depending on whether ProM is assessed in laboratory versus natural settings.

We also found that correlations between episodic ProM versus RetM and personality factors were comparable. Only Openness correlated significantly with RetM and the correlation was also very small, *r* = .09. Correlations between personality and RetM have been reported previously but they were generally small and found in some studies but not in others. Consistent with our findings, previous studies often reported that RetM correlates positively with Openness and negatively with Agreeableness, although such correlations tend to be small and often not significant even in large sample studies [17].

With respect to symptoms of psychopathology, we generally found very small negative correlations between both episodic ProM and RetM. Specifically, episodic ProM was weakly negatively correlated with Obsessive-Compulsive symptoms, Depression, Anxiety, Hostility, Phobic Anxiety, Paranoid Ideation, and Psychoticism. These correlations were similar to those found for RetM. These findings suggest that the negative effects of psychopathology symptoms are general rather than specific to only some of the symptoms. Nevertheless, however, they are not inconsistent with a suggestion that ProM correlations with symptoms of psychopathology may be very specific. For example, larger effects for checkers but smaller effects for non-checkers, within obsessive-compulsive subclinical and clinical disorders [29].

We also examined the correlations between episodic ProM and participants' sex. Although we observed a small correlation between participants' sex and RetM favoring women (*r* = .15), the correlation between participants' sex and episodic ProM was nearly zero and not significant. Moreover, we found no appreciable differences between correlations for men and women, for measures of ProM, RetM, cognitive abilities, personality, and symptoms of psychopathology.

Despite our large sample size and use of reliable continuous measures of ProM, our findings are limited to episodic even-cued ProM, assessed in laboratory settings, and to undergraduate students–a common limitations of many previous studies on relationships between personality, psychopathology and ProM and RetM. They may or may not generalize to other prospective memory subdomains, naturalistic settings, or other populations. Future studies will need to examine generalizability our out findings to other ProM subdomains, other settings and other populations.

## Conclusions

Our investigation was motivated by contradictory findings about the relationship between ProM and fundamental cognitive functions, personality, and symptoms of psychopathology. We examined these correlations in a large sample study, used reliable continuous measures of episodic ProM, and compared them to the correlations with RetM. Contrary to numerous prior findings, episodic ProM correlated with fundamental cognitive functions including processing speed, working memory, crystallized intelligence, fluid intelligence, and RetM. Although the pattern of the correlations was partially different for ProM versus RetM, overall these cognitive functions explained a similar proportion of variance in both ProM and RetM. Consistent with our prior meta-analysis and prior studies, we found that episodic ProM is largely unrelated to the Big Five personality factors and that ProM and RetM are similarly related to the Big Five. Only Openness was significantly correlated with both ProM and RetM. Similarly, the symptoms of psychopathology were weakly negatively correlated with ProM and RetM. In summary, our results show that cognitive factors were far more influential than non-cognitive personality and psychopathology factors in explaining both ProM and RetM performance. Importantly, our results suggest that the previous studies failed to find these relationships because of methodological issues including the use of inefficient, unreliable, binary measures of ProM; ceiling effects; and small sample sizes resulting in lack of statistical power.

Our findings are specific to episodic event-cued ProM assessed in laboratory settings. They may or may not generalize to other ProM subdomains or to a natural setting. Our previous research shows that it would be hazardous to assume that ProM correlations with cognitive functions, personality, and symptoms of psychopathology are invariant across ProM subdomains and assessment settings. For example, we found that age declines in episodic event-cued ProM are much larger than age declines in event-cued vigilance/monitoring [2]. Similarly, we found that ProM relationships with personality varied by ProM subdomain [17]. Moreover, as discussed above, personality factors may be far more influential in natural settings as well as for personally important ProM tasks [17,46].
